# A route map for the patients journey

**DOI:** 10.1186/1750-1172-7-S2-A40

**Published:** 2012-11-22

**Authors:** Paola Pierri

**Affiliations:** 1 ALD Life, London, SE22 0RG, UK

## Background

The Route Map for Adrenoleukodystrophy (ALD) and Adrenomyeloneuropathy (AMN) is a user led research project to create a repository of information and knowledge about two specific rare diseases. This Route Map provides a comprehensive resource to be used by patients, families and health and social care professionals.

From our survey we know that more that 60% of our patients with ALD or AMN are not receiving enough information and often the information they receive is not in an easy and patient friendly language. We also know that this situation is quite common among rare diseases.

## The aims and the approach

The aims of the Route Map (RM) have been:

• Improving the quality of care for patients with ALD and AMN

• Empowering patients and their families, expressing the potential contribution they can give to the NHS system

• Improving the quality of the relationship between patients and specialists

• Improving awareness of the conditions

We have developed the RM using a patient centred approach. This has meant that patients have been involved since the beginning and in different phases of the project to co-design the RM. NHS professionals have also been involved in the design process.

We have talked with patients to develop the questionnaires to make sure that we were asking the right questions; involved patients in focus groups to discuss openly and collectively about some general and some more specific issue; explored things from a different angle starting from the patient experience perspective of the care they have received.

This has made possible to derive knowledge and create deep understanding of complex processes; starting from personal stories we have been able to design the patient’ journey map.

## Conclusion

As the final output of the project we have developed an online practical and cost-effective platform with the main information about the care for patients with these conditions [http://www.ald-amn.info].

This platform will improve the access to information on health and social care services for individuals and families with rare conditions.

We think the RM will have the potential to play a key role in personalised care planning for people with these conditions. Moreover the co-design approach has been a learning process for both patients and NHS professionals.

Our experience and the things we’ve learned (what worked and what didn’t work) are the legacy that we would like to share with everyone that is starting the same journey.

